# Increased Physical Activity in Preparation for a Women-Only Mass Participation Sport Event: A Framework for Estimating the Health Impact

**DOI:** 10.3390/ijerph17010098

**Published:** 2019-12-21

**Authors:** Jelle Schoemaker, Simon van Genderen, Willem I. J. de Boer

**Affiliations:** 1 Sports & Exercise Studies, Sports & Economics Research Centre, HAN University of Applied Sciences, 6525 AJ Nijmegen, The Netherlands; Simon.vangenderen@han.nl (S.v.G.); willem.deboer@han.nl (W.I.J.d.B.); 2 Department of Economics and Business, Groningen University, 9747 AJ Groningen, The Netherlands

**Keywords:** running, vigorous physical activity, public health

## Abstract

Mass participation sporting events (MPSEs) are increasing in popularity. However, little research exists into the potential value of these events for improving public health by enhancing physical activity (PA). The aim of this study is to estimate the health impact of increased physical activity as a result of preparing for an MPSE. Participants of a mass participation women-only running event were asked if they performed additional PA in preparation of the event, including the length (weeks) and intensity (min per week). Additionally, self-reported change in health status was evaluated. Based on these results, we have developed a framework for estimating the cumulatively gained quality adjusted life years (QALYs) and monetary value thereof. Of the respondents (*N* = 468; mean age 42.3 ± 11.9 years), 32% performed additional vigorous PA in preparation of the event, with an average of 63 min per week over 8.8 weeks. Performing additional vigorous PA significantly improved the odds of self-rated health. The estimated total health impact of participants preparing for the Marikenloop was 6.6 QALYs gained with a corresponding monetary value between EUR 133,000 and EUR 532,000. We believe our health impact framework helps to understand that MPSEs can be a notable part of the public health domain.

## 1. Introduction

Over recent decades, the number of people with lifestyle-related health problems has increased dramatically [1]. Worldwide, 54% of the population is overweight and 19% is obese [2]. This leads to shortened life expectancy, reduced quality of life and higher health care cost [3]. Therefore, improved health is an eminent political goal for many communities and governments [4]. Physical activity (PA) decreases the risk of many chronic diseases and contributes to a long and healthy life [5]. The World Health Organization (WHO) has formulated a general PA guideline for the adult population to maintain or improve health [6]. To meet this guideline, adults should perform vigorous activities, such as running and jogging [7], at least 75 min a week. Several studies demonstrate that increasing PA can contribute to an improvement of an individuals’ life expectancy [8,9]. The impact of a specific amount of increased PA on improved health in terms of quality-adjusted life years (QALYs) has been studied much less. QALYs are measures of the health benefits that combine duration and quality of life and are used in the economic evaluation of medical intervention. Beale et al. (2007) estimated that increasing the amount of moderate PA by 30 min each week over one year might lead to an increase of 0.0106768 QALYs [10]. This means that one additional minute of PA amounts to an increase of 0.0000074 QALYs (based on 12 months of 4 weeks). Several studies have used this outcome to estimate the health benefits of PA [11,12].

Mass participation sporting events (MPSEs) are open entry events that, in many cases, require vigorous physical activity, such as running, cycling or swimming [13]. In recent decades, MPSEs have become increasingly popular in many countries [14] and are also considered to have a great potential to contribute to the health of participants [15,16]. Weed et al. (2012) argue that community-based events, like MPSEs, where people can participate individually, could create the desire to become more active, even for people that are currently not active [17]. Empirical evidence shows that MPSEs can influence participants’ attitude and behavior towards PA during the event preparation, event participation, and post-event reflection [18,19,20,21]. Teixeira et al. (2012) highlight the importance of autonomous and intrinsic motivations in long-term PA [22]. In addition, participation in sports is closely linked with intrinsic motivations such as enjoyment and achievement [23,24]. Some studies found that a significant number of the participants were not sufficiently active before preparing for the MPSE and became more active both before and after the MPSEs [13,23,25,26]. Moreover, Stevinson et al. (2013) found that initial non-runners participating in a weekly-organized MPSE reported more health-related benefits [27].

Since participating in an MPSE often requires a severe effort from the participants, many of them prepare for it by performing vigorous physical activity in anticipation of the event [13,23]. When a participant performs more PA than he or she would have done without participated in the event, it may improve his or her health. However, the existing body of knowledge around MPSEs is mainly concentrated on the effects on sport participation, life satisfaction or participants spending, but far less on the direct health effects of preparation and participation [21]. MPSEs may be particularly valuable for society because they can encourage people that tend to have lower physical activity levels to be active [27]. For health interventions, studies have estimated the (cumulative) QALYs gained, and the monetary value thereof, to determine the outcome and calculate the cost-effectiveness [28,29]. However, a similar conceptual framework for estimating the health effects of an MPSE does not exist. 

The purpose of this study is to examine to what extend preparing for an MPSE, in this case a woman-only running event in the Netherlands, contributes to performing more PA. We will also assess the association of additional PA with self-reported health states, with the hypothesis that doing additional PA in training for an MPSE will improve the participant’s subjective well-being. Finally, we will provide a novel framework for an MPSE’s ´health impact´, which we define as: the monetarized value of the additional amount of PA performed by participants of the MPSE who were initially physically inactive. Our research question is: what is the health impact of a women-only MPSE in terms of additional PA performed during the preparation of the event, the estimated QALYs gained and the monetary value thereof?

## 2. Materials and Methods

The MPSE of interest for this study was the 2017 Marikenloop, held in the city of Nijmegen, the Netherlands. The Marikenloop is an annual women-only running event of 5, 7.5 and 10 km. Because this event has no male participants and relatively short distances, it has a relatively low threshold to participate, especially for women that do not participate in sport or exercise regularly. Women are less active than men in the Netherlands [30] and worldwide [13], and therefore the Marikenloop is a highly relevant object for this study.

### 2.1. Data Collection

A cross-sectional online-survey was conducted the week after the event. The 7304 participants who completed the race received an invitation by e-mail including a link to complete an online survey. Informed consent for this email was obtained upon registration and participation in the survey was voluntarily. Participants could complete the questionnaire anonymously and a reminder was sent to all participants after 7 days. No questions were included that would harm the reliability of the respondents or make them retraceable.

### 2.2. Measures

Items from the Short Questionnaire to Assess Health-Enhancing Physical Activity (SQUASH) [31,32] was used to ask participants to indicate their current habitual activity level for vigorous PA. They were also asked whether or not they performed more (i.e., additional) training or sport activities in order to prepare for the event, compared to the (hypothetical) case that the given MPSE would not have been organized. Subsequently, participants who indicated to have performed additional vigorous PA were asked about the duration (number of weeks) as well as the severity (min per week) of the preparation period and to indicate the amount of training or sports (on a vigorous activity level) they would have performed in that same period, if there would not be an event organized. 

Following the Short Form Health Survey (SF-36), participants were asked to rate their general health status and indicate how they would rate their current health compared to the period before they started their preparation for the event, on a 5-point scale [33]. Finally, demographic- and running-related items were added to the survey; comprising age, height, weight, most common training type (individually or in a group) and the running distance at the event.

### 2.3. Data Analyses

For each participant of the Marikenloop, the amount of additionally performed vigorous PA was calculated by multiplied the average preparation period (in weeks) with difference between the actual weekly performed amount of PA (min per week) and the specified average amount of PA without the event being organized. A dummy was created to distinguish participants who initially would not have met the WHO guidelines for vigorous activity (1) from those who did comply with these guidelines (0). Regarding the subjective health status, two categories were distinguished as the self-rated health was either improved (categories much better or somewhat better) or not improved (all other categories). 

After checking the normality of the data, comparisons were made on the perceived health status and change in health status between participants who performed additional vigorous PA and those who did not, using independent t-tests. We used Chi-square tests to analyze to what extent the amount of additional vigorous PA of a participant is associated with a perceived health change. Binary logistic regression was used to investigate the relationship perceived health changes and several background variables, such as age, BMI, distance completed during the event and additionally performed vigorous PA categories. All analyses were performed with PASW Statistics 23 (SPSS, Chicago, IL, USA).

### 2.4. Framework for Estimating the Health Impact

**Figure 1:**
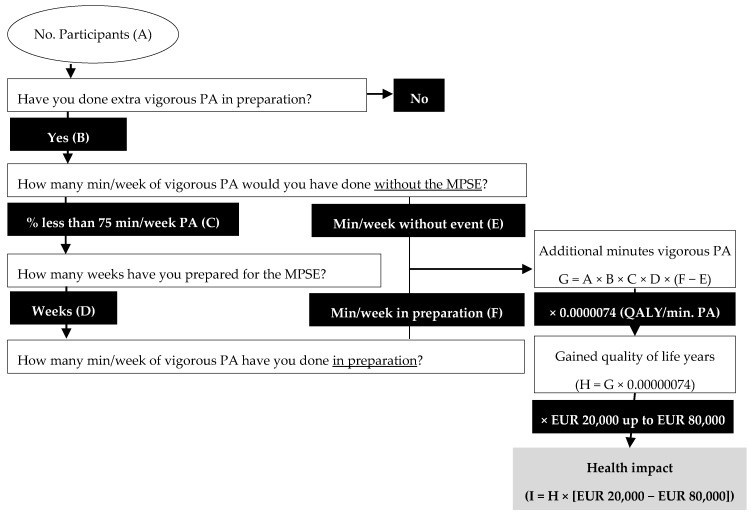
Theoretical framework of the health impact of an MPSE.

We used the framework of Mosely (2018) to relate the additional PA to the health impact of preparing for an MPSE as presented in Figure 1 [11]. An MPSE attracts a number of participants (A), of whom a percentage (B) has specifically prepared for the event by doing more vigorous PA than usual. Of this group (A × B), a certain fraction (C) would not have met the WHO guidelines for (vigorous) PA, without preparing for the event. Importantly, we make the assumption that participants who perform enough PA according to the WHO health guidelines are unlikely to improve their health (much) by further increasing their PA. Consequently, their health impact is, admittedly arbitrarily, set at zero. For the group of initially inactive participants, we determined how much additional vigorous PA they performed by multiplying the number of weeks of preparation (D) by the difference of average amount of PA, in min per week, they have performed during preparation (F) minus the amount of PA they would have performed without the event (E). The outcome (G = A × B × C × D × (F – E)), is the total number of additional minutes of vigorous PA performed in preparation of the MPSE by individuals that were initially inactive. Multiplying this outcome by the amount of QALYs gained for every additional minute of vigorous PA (0.0000074, from Beale et al. [10]), results in the estimated the number of QALYs gained in association with preparing for participating in the MPSE (H). Finally, to monetarize this health effect, we multiply this outcome (H) by a reference value of one QALY. In the Netherlands, both the Health and Society Counsel (RVS) and the National Health Care Institute (NZI) consider EUR 80,000 as the reference value for the maximum amount of money that may be spent on a new treatment per QALY, while interventions costing EUR 20,000 per QALY (or less) are seen as cost-effective [34,35]. Other institutions, such as the United Kingdom’s National Institute for Health and Care Excellence (NICE) also use thresholds in the range of EUR 20,000 [36].

### 2.5. Study Sample

A total of 510 participants filled in the survey (7% response rate). Due to 42 incomplete cases, the final sample consisted of 468 questionnaires. This exceeds the minimum number of 365 needed for a statistically representative sample for the Marikenloop’s total population size of 7304 (for a 5% margin of error, 95% confidence level). Table 1 shows the descriptive statistics of the survey. Participants were on average 42 years old and had a mean body mass index of 23.4. Almost a third could be classified as overweight (29%) or obese (3%). Almost half (45%) of the respondents ran the 5 km distance, 22% the 7.5 km and 33% the 10 km. This is consistent with the distribution of the full population of the Marikenloop in terms of distance ran (49% 5 km, 24% 7.5 km, 27% 10 km), but in terms of age there are more older participants in the sample (the average age is 37 years for all participants of the Marikenloop). No information about weight, length, training or health status is available for the total population of this MPSE. As for the general adult female population, the current sample contained a comparable amount of overweighed participants opposed to the general female Dutch population (32% vs. 30%), but yielded fewer obese participants (3% vs. 14%). For this reason, the current results should be interpreted with caution and cannot be generalized for the overall Dutch female population.

More than 60% of the participants of the Marikenloop did their training individually. Only 4% of the participants categorized their current health as either ‘poor’ or ‘fair’, whereas over 40% had a self-perceived health of ‘very good’ or ‘excellent’. Participants performed vigorous PA on an average of 2.4 times/week for 52 min, resulting in 130 min on average per week.

## 3. Results

First, we made a distinction between participants that did and those that did not perform additional training in preparation of the event. Almost a third (32%) of the participants performed additional vigorous PA in preparation of the Marikenloop. The preparation time for the MPSE for this group of participants was on average 8.8 weeks. Table 2 shows that participants with additional vigorous PA were significantly younger (2.4 years, *p* < 0.05), more likely to train individually (*p* < 0.05) and less likely to participate in the 10 km event (*p* < 0.001). However, no significant differences were found in BMI and self-rated health among participants. Of the participants that performed additional vigorous PA, 5% claimed to have a ‘much improved’ health, whereas around 37% had a ‘somewhat improved’ health, compared with their health status before they did extra PA.

The results of the logistic regression revealed that regarding the additional vigorous PA, the odds ratio for reporting an improved health is 2.49 (*p* < 0.01) for individuals performing more than 450 min of additional vigorous PA than those who perform less. This outcome is also robust when corrected for age, BMI and distance completed. However, there was no significant difference in the reported change in health between participants that initially did and those that did not meet the WHO guidelines for vigorous activity.

### Health Impact Marikenloop

**Figure 2:**
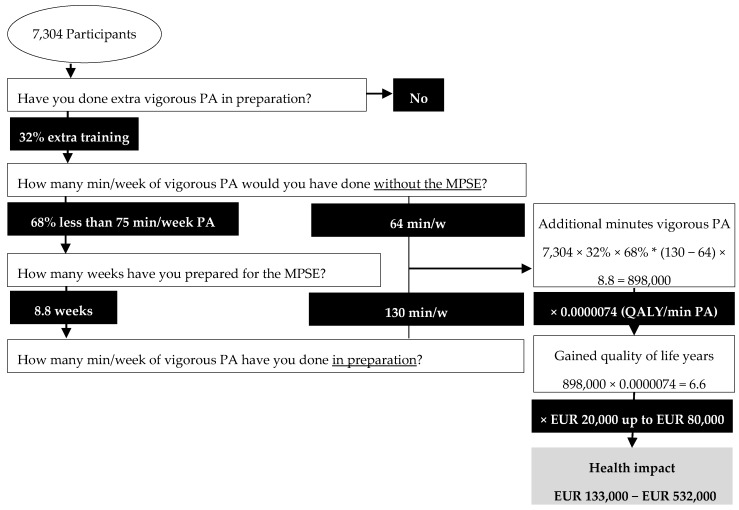
Health impact of the 2017 Marikenloop.

With the assumption that our research sample is representative for the whole population, the framework for health impact plays out as follow for the Marikenloop (see Figure 2). Among the 7304 participants (A) of the MPSE, 32% (B) performed additional vigorous PA in preparation for the event, of whom 68% (C) did initially not comply with the WHO guidelines for vigorous PA. This amounts to 1589 (A × B × C) initially inactive participants, performing additional PA for on average of 8.8 weeks (D) in preparation for the event. Without the event, this group would have performed on average 64 min of PA per week (E), whereas with the event their PA increased to an average of 127 min per week (F). This leads to an average of 63 min (F − E) of additional vigorous PA per week and 565 min (D × (E − F)) in total, for participants who performed extra training in preparation for the event. The total cumulative amount of additional min of vigorous PA for the 1589 inactive participants is then 898.000 (G). This in turn corresponds to an estimated 6.6 gained QALYs (H) and a monetary value (I) of EUR 133,000 up to EUR 532,000, depending on which reference value per gained QALY is that is taken (NICE for the lower estimate; WHO or NZI for the upper estimate). Hence, we estimate the health impact value of preparing for the 2017 Marikenloop between EUR 133,000 and EUR 532,000.

## 4. Discussion

For the 2017 Marikenloop, 32% of the participants performed additional PA in preparation of the event. This finding is well below the outcomes of studies on similar events. Derom et al. [23] reported that half of the participants of a mass participation cycling event became more active in the preparation period. The study of Bowles et al. [25] revealed that 68% of the participants increased their level of PA by preparing for the 20 to 50K cycling event, while Lane et al. [26] found out that 85% of the participants of a women-only 10K run did additional PA in preparation of the event. Probably, running a 5 to 10K race, like the Marikenloop, requires less preparation than these other events researched. On the other hand, 21% (1589 participants) of our study sample indicated that they initially did not meet the threshold of 75 min of vigorous activity. By contrast, only 12% of the triathlon participants [13], and 4% of the cycling participants [25] were not sufficiently active before preparing for the event. However, those studies used the minimum of 150 min of moderate activity a week to determine who was not sufficiently active. Although the threshold of 75 min of PA could indicate a possible bias, it could also be that the Marikenloop is more accessible for people that are less active.

In our framework, we have used the outcomes of the study of Beale et al. [10] to translate the additional amount of PA to a volume of QALYs gained. By doing this we adopt the assumption of a linear relationship between the PA and QALYs. However, this assumption seems too bold, since decreasing returns to scale seem very likely [10]. In our analysis, we have addressed this by assuming no health effects for participants that initially already met the WHO norm. Although this adjustment likely prevents our framework from potentially highly overestimating the health impact of an MPSE, the outcomes may still be an over- or an underestimation of the ‘real’ amount of QALYs gained. For running a significant positive effect on, e.g., mortality has been demonstrated [37], but no dose-effect could be found (i.e., the amount of running did not seem to matter). In addition, in our study we did not find any statistical differences in self-perceived health change, between the group that initially did meet the WHO threshold and the group that did not. Additional studies are needed to shed a clearer light on the causal health effects of participating in MPSEs, as well as the underlying mechanism that determine an MPSE potential outcome [38].

Similar to several other studies we offer a framework on the possible gains in PA caused by an MPSE [13,23,25,26], but unlike these studies, we quantify the amount of additional min of PA performed in preparation for the event. Our study therefore contributes to the literature to identify an MPSE, and in particular the preparation period, as an opportunity to enhance health.

Our results show that participants who are young or who are running short distances were significantly more likely to do more additional PA in the preparation period. This contrasts with earlier research showing that those who did the longer distance (259 km vs. 83 km cycling) significantly increased their physical activity, whereas those in the shorter distances were less likely to change their PA habits [23]. Future studies are necessary to see what potential target groups are to improve health through preparing for and participating in an MPSE. In comparison to more traditional health interventions, an MPSE does not have a primary objective to sustain or improve aspects of health. Motives to participate are likely intrinsically driven in terms of fun and liking the sport activity on itself [18].

Public health policies may investigate MPSEs as a low-cost, low-barrier intervention for health improvements for certain target groups. These include participants and potential participants who are young or who are interested in running short distances. Perhaps general practitioners (GPs) and lifestyle coaches could stimulate physically inactive individuals to participate in running events by giving ‘prescriptions’ or policy makers could combine MPSEs with other instruments like routes, water taps, healthy school initiatives and/or community programs [15].

The framework that we present provides a novel perspective on measuring PA-related health improvements of MPSEs, because it delivers outcomes that are in line with other health (QALYs) and economic measures (monetary values). In addition, this framework is simple to interpret and apply. By explicitly showing the health benefits of MPSEs in terms of health impact, organizers and financers of these events can make their societal value added visible, while public health officers may consider stimulating participation of MPSE as a potential low barrier, low cost health intervention. This framework may enhance the visibility of health effects of MPSEs, in addition, but also as a counterbalance, to the often-used ´economic impact´ of sports events [39].

Our research has several limitations. First of all, this study can only address association and not prove causality between health and additional PA. In addition, our study uses self-perceived indicators of health and PA. We are aware that such measures are prone to bias and therefore suggest further validation of the current instrument in terms of content and predictive validity. Contrarily, the health items within the SF-36 are widely used and validated.

By using the WHO guidelines for vigorous activity, we excluded moderate or low intensity PA. As discussed before we do not know if preparing for the event led to a crowding out effect in lower intensity activities or if participants complied with the guidelines by doing 150 min a week of moderate activity. We also did not look into the negative health effects and assumed that our sample was representative for the whole research population. Moreover, these results are based on a women-only running event. This and other characteristics of the Marikenloop may dramatically lower the barriers for entering the event, particularly among women who are not (sufficiently) physically active.

Although the health impact framework itself is independent of the event context, the presented outcomes may not be applicable for other types of MPSEs. Future research should address these shortcomings by investigating other types of MPSEs and asking participants about their other activities, injuries and do a follow up to see if the health improvements last. Finally, this study also uses several assumptions including the estimate for QALY/min, linear health returns and the threshold for QALYs. As mentioned above, these assumptions need to be studied in future research.

## 5. Conclusions

In this study, we found that about one third of all participants at the women-only running event Marikenloop performed additional PA in preparation of this MPSE. Of the participants that did additional PA in preparation of the event, a large proportion would not have met the WHO guideline of at least 75 min of vigorous PA per week without the event. Thus, an MPSE has the potential to increase the amount of vigorous PA for participants in the preparation period towards the event. Furthermore, this group reported an improved state of health. The odds of reporting improved health were significantly higher for the half that performed the most additional vigorous PA, compared to the half that did the least additional PA in preparation. We proposed a new framework to for the health impact of the event, by estimating the cumulative number of additionally performed minutes of vigorous PA and translating that to the number of QALYs gained and finally monetary value. For the Marikenloop in total, we estimated that participants who initially did not meet the WHO norm, performed an estimated total of 898,000 additional min of vigorous PA in preparation of the event. In our framework, this is equivalent to 6.6 QALYs gained and represents a health impact of EUR 133,000 up to EUR 532,000. The health impact of an MPSE may complement the event’s economic impact and help justify investments from public resources. This makes MPSEs effectively part of the public health equation.

## Figures and Tables

**Table 1:** Descriptive statistics.

Study Variables	*N*	Minimum	Maximum	Mean	SD
Age (years)	468	13	74	42.3	11.9
Weight (kilograms)	468	43	120	67.5	10.1
Length (meter)	468	1.50	1.90	1.70	0.06
BMI	468	15.9	38.5	23.4	3.2
Obesity (yes/no)	468	0	1	0.03	0.2
Distance (kilometers)	468	5	10	7.2	2.2
Individual training (yes/no)	468	0	1	0.62	0.5
Current health status (1–5)	468	1	5	3.5	0.7
Times a week PA (preparation)	468	0	7	2.4	1.0
Min PA/time (preparation)	468	0	220	52.1	23.3
Total min PA (preparation)	468	0	420	130	77.1
Extra training preparation (yes/no)	468	0	1	0.32	0.5
Extra training weeks	151	1	30	8.8	5.4
Times a week PA (without event)	151	0	4	1.5	1.0
Min PA/time (without event)	151	0	120	41.6	19.0
Total min PA (without event)	151	0	240	64.3	56.3
Inactive without event (yes/no)	151	0	1	0.68	0.5
Min additional PA/week	151	2	300	63.3	43.5
Min additional PA total	151	10	3000	565.6	545.5
Health change (−2–2)	151	−1	2	0.45	0.64
Health improved (yes/no)	151	0	1	0.42	0.50

**Table 2:** Differences between participants who do or do not extra train in preparation of the event.

Study Variables	Extra Training (*N* = 151)	No Extra Training (*N* = 317)	Significance
Age	41 (11)	43 (12)	0.040
BMI			
Underweight	5 (3%)	7 (2%)	0.400
Normal weight	95 (63%)	224 (71%)	
Overweight	46 (31%)	77 (24%)	
Obesity	5 (3%)	9 (3%)	
Health			
Poor	0 (0%)	1 (0%)	0.569
Fair	6 (4%)	12 (4%)	
Good	87 (58%)	171 (54%)	
Very good	50 (33%)	103 (33%)	
Excellent	8 (5%)	30 (10%)	
Condition training			
Group	44 (29%)	136 (43%)	0.004
Individual	107 (71%)	181 (57%)	
Distance			
5 km	80 (53%)	134 (42%)	0.003
7.5 km	37 (25%)	61 (19%)	
10 km	34 (23%)	122 (39%)	
Preparation (min/week)	128 (69)	131 (81)	0.632
